# Psychometric Properties of the Persian Version of the Teasing Questionnaire 23

**DOI:** 10.3389/fpsyg.2021.664736

**Published:** 2021-04-15

**Authors:** Ali Ebrahimi, Mojtaba Elhami Athar, Mitra Hakim Shooshtari, Hossain Karsazi, Eric A Storch

**Affiliations:** ^1^Department of Clinical Psychology, School of Behavioral Sciences and Mental Health (Tehran Institute of Psychiatry), Iran University of Medical Sciences, Tehran, Iran; ^2^Department of Psychiatry, School of Behavioral Sciences and Mental Health (Tehran Institute of Psychiatry), Iran University of Medical Sciences, Tehran, Iran; ^3^Faculty of Psychology and Educational Sciences, University of Tehran, Tehran, Iran; ^4^Department of Psychiatry and Behavioral Sciences, Baylor College of Medicine, Houston, TX, United States

**Keywords:** internal consistency, factor structure, persian version, teasing questionnaire, TQ-R

## Abstract

The current study was a cross-sectional research and aimed to investigate the factor structure, internal consistency, and validities of the Persian version of the Teasing Questionnaire-Revised (TQ-R). Forward and backward translations of the TQ-R were performed; face and content validities were determined based on comments by a sample of psychology students and specialists. Using the cluster sampling method, 290 participants were recruited, and 201 valid data (*M*_age_ = 23.53, SD = 3.53, 64.2% men) were analyzed. The factor structure was assessed by confirmatory and exploratory factor analysis (EFA). The result of the confirmatory factor analysis(es) did not confirm the proposed three, four, and five-factor models. EFA revealed four factors with 23 items, explaining 52.03% of the total variance. The internal consistency of the Persian version of Teasing Questionnaire 23 was in the excellent range (α = 0.92), and its expected associations with external correlates (e.g., depression and anxiety) supported the measure’s convergent validity. The findings indicated that the Persian version of the TQ-R has sound psychometric properties and can be used as a valid and reliable tool in research and clinical outcomes.

## Introduction

Teasing refers to a verbal or non-verbal provocation delivered directly or indirectly that includes a comment on something relevant to the victim (Keltner et al., 2001). It is a common, complex, and potentially distressing form of human interaction (Kruger et al., 2006; Zhou and Luo, 2015). Teasing is one of the most common forms of bullying, which is defined as aggressive, intentional, abusive, and repetitive behavior, causing harm and suffering for another individual (Storch et al., 2004).

Numerous studies have indicated a relationship between experiences of being teased during childhood and adult psychopathology (Roth et al., 2002; Storch et al., 2004; McCabe et al., 2010; Benas and Gibb, 2011; Stitt et al., 2015; Gregg et al., 2016; Zlomke et al., 2016; Szwimer et al., 2020). Being the victim of teasing and related experiences like verbal and emotional abuse has been associated with eating disorders, depression, anxiety, borderline personality disorder, substance abuse, cognitive bias, low self-concept, and low self-esteem (Storch et al., 2004; Kostanski and Gullone, 2007; Stitt et al., 2015; Zlomke et al., 2016; Lund and Ross, 2017). In addition, childhood teasing has been associated with increased symptoms of anxiety and depression in students (Roth et al., 2002; Storch et al., 2004; Strawser et al., 2005), non-clinical adults (Muris and Littel, 2005; Faith et al., 2008), and patients with anxiety disorders (McCabe et al., 2003, 2010). Teasing victims often report feelings of exclusion from society, loneliness, and anxiety (Zlomke et al., 2016). Furthermore, there is a relationship between childhood teasing and adult psychopathology based on the type of teasing. For example, when one is teased during childhood due to performance, fear in adulthood becomes more related to negative evaluations (Storch et al., 2004). Thus, given the association between childhood teasing and adulthood psychopathologies, the development of reliable tools for assessing childhood teasing experiences is of utmost importance.

Several self-report measures have been developed to measure teasing during childhood (Thompson et al., 1991, 1995; Storch et al., 2004). However, most of these questionnaires focus only on evaluating teasing due to physical appearance while overlooking other domains. To address this issue, Roth et al. (2002) designed the Teasing Questionnaire (TQ) to evaluate teasing recall from several domains. However, initial psychometric studies revealed that the TQ best measured one domain of teasing but not various domains of the experience of being teased (Roth et al., 2002; Storch and Ledley, 2005).

To provide a tool that could assess multiple domains of being teased, Storch et al. (2004) developed the Teasing Questionnaire-Revised (TQ-R). Compared with the TQ, the TQ-R is different in terms of content (several new items have been added to the revised form). In addition, the factor structure is changed, and the TQ-R considers five factors instead of one, but the main scoring structure is preserved. As a result, based on a five-factor model using a structural equation model, the derived 29-item measure assesses childhood experiences of being teased based on Performance, Academic Issues, Social Behavior, Family Background, and Appearance. Previous studies have suggested that TQ-R has good psychometric properties (Storch et al., 2004; Strawser et al., 2005; Faith et al., 2008; Gros et al., 2012).

Teasing has been studied in different cultures and societies, and measures to assess teasing experiences have been developed. Faith et al. (2008) examined the psychometric properties of the TQ-R in a community sample of adults within diverse ethnicities. Their results proposed a three-factor model consisting of Academic, Social, and Appearance. The Cronbach alpha ranged from 0.83 to 0.90. Similarly, Tian-mei (2009) investigated the psychometric of the Chinese version of the TQ-R among middle school students. Twenty-eight items were loaded on five factors; Cronbach alpha of 0.90 was reported for the TQ-R total score alpha coefficients larger than 0.65 reported for the subscales.

In Turkey, Mutlu and Yilmaz (2018) examined the psychometrics of a Turkish version of the Child–Adolescent Teasing Scale (CATS) among Turkish children. A four-factor model was proposed for the measure, and reliability coefficient scores ranged from 0.56 to 0.93.

Similar to other countries (Gadekar, 2016; Zuba and Warschburger, 2017; Watanabe et al., 2019), teasing is a widespread phenomenon in Iran’s society and schools; however, a suitable measure for assessing teasing experiences is not yet available in Iran. Thus, there is a clear need to translate and evaluate established measures of teasing for researchers to investigate teasing experiences and their consequences in Iranian society.

In the current study, we first provide the forward and backward translation of TQ-R. Then, we will examine the face and content validity of TQ-R. Next, we examine the factor structure and the reliability of TQ-R in a community (*n* = 290) sample. To test the factor structure of the TQ-R, confirmatory factor analyses (CFAs) and exploratory factor analysis (EFA) will be performed. We also calculate reliability indices (Cronbach’s α and mean inter-item correlation) values to examine the reliability of the Persian TQ-R scores. Finally, to test the TQ-R’s convergent validity, we considered variables that have been shown to be correlated with TQ-R (e.g., Anxiety and Depression). We hypothesized that TQ-R would be associated positively with Depression and Anxiety.

## Materials and Methods

### Participants

The participants of the current study were 18–35-year-old university students between December 2018 and March 2019. Approximately 10–20 subjects should be considered for each item of any questionnaire (Schumacker and Lomax, 2015). Thus, since the number of items in the questionnaire was 29, with 10 participants for each item, we recruited 290 students for our sample, using the cluster sampling method. However, after screening the data for outliers and missing values, 201 valid data (*M*_age_ = 23.53, SD = 3.53, 64.2% men) were analyzed.

### Procedure

This study was a cross-sectional research and evolved from the research project affiliated with the School of Behavioral Sciences and Mental Health (Tehran Institute of Psychiatry), Iran University of Medical Sciences, Tehran, Iran. The ethical committee of the Iran University of Medical Sciences (code number IR.IUMS.REC 1394.94-05-121-27889) evaluated and approved this study. After corresponding with the original developer of TQ-R 29, Storch et al. (2004), we received the English version of the TQ-R. Forward and backward translation processes (Epstein et al., 2015) were then performed; the TQ-R was translated into Persian by two independent individuals fluent in English and Persian and back-translated by an English language expert. The authors matched the translated items with the original ones. To determine the face validity of the TQ-R, we recruited 10 students using the convenience sampling method and asked them to complete the measure and report any concerns, questions, or misunderstandings about the clarity of the sentences, response format, and/or sentence structure of the items. Based on the students’ feedback, we modified the problematic statements to make them more straightforward and transparent.

To examine the content validity, we implemented comments by three specialists in Clinical Psychology and two specialists in Psychiatry who were familiar with the teasing concept and the instrument; the sample was selected using the convenience sampling method.

Next, we started the data gathering process. The University of Tehran was selected as the first sampling unit. Some faculties were then selected, and finally, classes were randomly selected. The administration of the survey was conducted in the classroom on a regular school day during a 1-h session under the supervision of a specially trained research assistant (master-level student). Before conducting the study, the study aim was explained to the participants, and they were told that there was no obligation to participate in the study nor any compensation. After an agreement to participate and an assurance of confidentiality, an informed consent form was taken from the participants, and then, they were asked to complete the TQ-R, Beck Depression Inventory-II (BDI-II), and Beck Anxiety Inventory (BAI). The response rate was 69.31%. Inclusion criteria included being an undergraduate or graduate student and interest and willingness to participate in the study.

### Measures

#### Teasing Questionnaire-Revised

The TQ-R is a 29-item scale designed by Storch et al. (2004) to measure teasing memories during childhood. Responses are made on a 5-point Likert-type scale (0, “*I was never teased about this*;” 1, “*I was rarely teased about this*;” 2, “*I was sometimes teased about this*;” 3, “*I was often teased about this*;” and 4, “*I was always teased about this*”). Subscales of the TQ-R consist of Performance, Academics, Social Behavior, Family Background, and Appearance. Cronbach’s alpha coefficients for these subscales were 0.58, 0.84, 0.70, 0.48, and 0.78, respectively (Storch et al., 2004). Cronbach’s alpha and MICs for TQ-R and its factors in this study can be retrieved from Table 1.

**TABLE 1:** Descriptive statistics of TQ-R, BDI-II, and BAI variables.

Measures	Mean	Range	SD	Skewness	Kurtosis	α	MIC
TQ-R_total	11.94	0–64	13.32	1.525	2.055	0.93	0.36
Appearance	3.09	0–22	4.64	1.992	3.798	0.85	0.45
Social	4.07	0–22	5.08	1.629	2.152	0.85	0.42
Height	0.621	0–6	1.33	2.359	5.060	0.73	0.58
Academic	4.154	0–20	4.73	1.190	0.830	0.85	0.48
BDI-II	12.82	0–51	10.47	1.227	1.378	0.75	0.36
BAI	10.52	0–45	8.96	1.326	1.802	0.89	0.28

*TQ-R, teasing questionnaire-revised; BDI-II, beck depression inventory-II; BAI, beck anxiety inventory; SD, standard deviation; MIC, mean interitem correlation.*

#### Beck Depression Inventory-II

The BDI-II is a 21-item self-report measure for assessing depressive symptoms during the previous 2 weeks. Each item includes four choices, and patients choose the one that best describes the intensity of their depressive symptoms. Items are rated from zero to three in terms of intensity. Cutoff scores for BDI-II include none or minimal depression (0–13), mild depression (14–19), moderate depression (20–28), and severe depression (29–63). Factor analysis of the BDI-II indicated two factors, including the Non-cognitive and Cognitive-Affective factors (Beck et al., 1988b). Some research indicated three factors, including Cognitive, Somatic, and Affective (Manian et al., 2013). A reliability study of BDI-II on clinically depressed patients in Iran indicated excellent reliability (α = 0.91) (Stefan-Dabson et al., 2007). Cronbach’s alpha and MICs of BDI-II in this study can be retrieved from Table 1.

#### Beck Anxiety Inventory

BAI is a 21-item self-report measure wherein each item is rated from zero to three in terms of intensity of anxiety symptoms experienced during the previous week. The total score ranges from 0 to 63. Each item assesses one of the anxiety symptoms commonly experienced by clinically anxious people or those exposed to anxiety-provoking situations. BAI’s original version had excellent internal consistency (Beck et al., 1988a). The Persian version of BAI indicated good reliability (*r* = 0.72, *p* < 0.001), validity (*r* = 0.83, *p* < 0.001), and excellent internal consistency (α = 0.92), so it could be used in clinical assessment and research (Kaviani and Mousavi, 2008). Cronbach’s alpha and MICs of BAI in this study can be retrieved from Table 1.

### Data Analyses

In the current research study, SPSS 18.0 (Meyers et al., 2013) was used to perform descriptive characteristics of the study samples and measures. After screening the data for outliers and using the missing values analysis procedure in SPSS 18.0 with the expectation-maximization method, missing values were imputed, resulting in a sample size of 201.

To examine the construct validity of the Persian version of TQ-R, confirmatory and exploratory factor analyses were conducted through Lisrel 8.80 using the maximum likelihood estimator (Du Toit et al., 2001). Model fit was assessed using incremental fit index (IFI), the comparative fit index (CFI), and root mean square error of approximation (RMSEA). We considered an IFI and CFI equal to 0.90 and higher as an adequate fit and amounts higher than 0.95 as an excellent fit. In addition, an RMSEA equal to 0.08 or lower was considered an adequate fit, and values equal to 0.05 or lower were considered an excellent fit (Hu and Bentler, 1999). Next, the TQ-R scores’ internal consistency was examined using Cronbach’s α and MIC values. Data were reviewed and analyzed using SPSS version 18 and Lisrel 8.80 (Du Toit et al., 2001; Meyers et al., 2013).

Finally, to evaluate TQ-R’s convergent validity, Pearson correlation coefficients were examined between the TQ-R scores and variables of interest assessed by means of the BDI-II and BAI.

## Results

Confirmatory factor analyses were conducted to test the proposed five- (Storch et al., 2004), three- and four- (Faith et al., 2008), and five-factor models of the TQ-R (Gros et al., 2012). The results showed that none of the proposed models yield adequate fitness (Table 2).

**TABLE 2:** The goodness of fit indices for all CFA models of TQ-R.

Model	χ2	df	CFI	IFI	GFI	RMR	RMSEA
4 F—24 items (Faith et al., 2008)	638.3	203	0.93	0.90	0.78	0.067	0.104
3 F—15 items (Faith et al., 2008)	203.85	62	0.93	0.93	0.86	0.067	0.107
5 F—29 items (Storch et al., 2004)	899.04	289	0.93	0.93	0.74	0.066	0.103
5 F—21 items (Gros et al., 2012)	153	129	0.94	0.94	0.82	0.055	0.100

*df, degrees of freedom; CFI, comparative fit index; IFI, incremental fit index; RMR, root mean square residual; RMSEA, root mean square error of approximation; CFA, confirmatory factor analysis.*

As a result, we conducted EFA on the 29 items of the TQ-R. The result of Kaiser–Meyer–Olkin (KMO = 0.895) indicated the sampling adequacy for the study sample. In addition, Bartlett’s test of sphericity (chi-square = 6886.608, *p* < 0.001) suggested using factor analysis.

At first, since the kurtosis and the skewness of items 3 (3.35 and 11.68), 11 (4.03 and 25.78), and 23 (3.37 and 11.50) were higher than the range proposed by Kline (2015) (±3 skewness and ±8 kurtosis), these items were eliminated.

**FIGURE 1:**
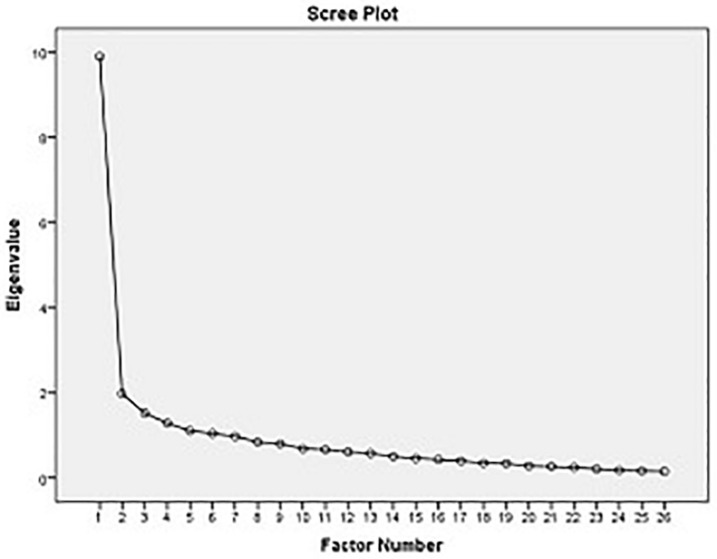
Scree plot to determine the number of factors.

In an EFA, the obtained results based on the Kaiser criterion led to a six-factor solution, which explained 54.539% of the overall variance, and the results based on the scree plot (Figure 1) led to a four-factor solution. Since the research resources recommended solutions with a lower number of factors (Meyers et al., 2013; Kline, 2015), and because the scree plot showed a four-factor solution, the EFA was redone by fixing the number of factors to 4. This time, the four identified factors defined 49.224% of the overall variance in the factor analysis.

Items with a factor loading lower than 0.4 (items 12, 14, and 19) were eliminated. Thus, 23 items remained, which loaded on four factors, explaining 52.034% of the variance, with factor loadings ranging from 0.435 (item 22) to 0.818 (item 7). In reviewing the resulted factors, item 22 was moved from factor 3 to factor 2, item 25 was also moved from factor 2 to factor 4, and item 26 was moved from factor 3 to factor 1 to facilitate the interpretation and labeling of the factors. Finally, the first factor, with six items, explained 14.435% of the variance. The second factor included eight items and accounted for 13.496% of the variance. The third factor consisted of five items and explained 12.637% of the variance. The fourth factor comprised of four items and explained 11.467% of the variance (Table 3).

**TABLE 3:** Factor analysis with varimax rotation method of the four-factor model of TQ-R (*N* = 201).

Factor Loading					
Item Number	Abridged Items	Appearance	Social	Height	Academic
7	Aspects of my appearance	0.81			
13	The way that I dressed	0.64			
5	Ethnic differences	0.60			
26	Wore eyeglasses	0.60			
6	The family did not have as much money as other kids	0.59			
8	Color or style of my hair	0.58			
29	I had a funny name	0.45			
4	Being nerdy		0.57		
17	Shy around other kids		0.56		
24	Fear of doing activities like swimming and camping		0.54		
18	Was not good at performance activities		0.54		
22	Cried a lot/acted like a baby		0.43		
9	Was not a very cheerful kid		0.48		
16	Was not very good at conversations		0.44		
1	Not good at sports		0.46		
27	Taller than other kids			0.76	
28	My height			0.62	
2	Excelled at school				0.76
15	Teacher’s pet				0.74
2	Volunteered to answer questions in class				0.57
21	Matured earlier than other				0.54
10	Studies a lot				0.55
25	Cared more about classes than sports				0.44
Eigenvalues		3.32	3.10	2.63	2.90
% of variance		14.43	13.49	11.46	12.63
Cronbach’s a		0.85	0.84	0.73	0.84

Next, CFA was conducted on the 23 items, including items loading on Appearance (seven items), Social (eight items), Height (two items), and Academic (six items) subscales to test the fitness of the model obtained from EFA. The results of CFA supported the four-factor model’s fitness (CFI = 0.96, IFI = 0.96, RMSEA = 0.075).

### Internal Consistency and Correlations Between the TQ-R Scores

Overall, Cronbach’s alphas for the TQ-R were 0.92 for the TQ-R total score and ranged from 0.73 to 0.85 for the subscales, which are in the acceptable to good ranges (see Table 1). Likewise, when relying on the MIC as an index of internal consistency, the TQ-R total score and factors scores indicate excellent internal consistency (Table 1). Significant zero-order correlations were found between TQ-R factor scores and the TQ-R total score. These correlations were as follows: *r*_Appearance__–__total_ = 0.86; *r*_Social__–__total_ = 0.90; *r*_*Height*__–__CU_ = 0.63, and *r*_Academic–__total_ = 0.81 (Table 4).

**TABLE 4:** Pearson correlation, mean, and SD of TR-Q and its subscales with anxiety and depression (*N* = 201).

	1	2	3	4	5	6	7
1. Total	–						
2. Appearance	0.86**	–					
3. Social	0.90**	0.73**	–				
4. Height	0.63**	0.55**	0.49**	–			
5. Academic	0.81**	0.51**	0.62**	0.43**	–		
6. Depression	0.43**	0.38**	0.41**	0.21**	0.35**	–	
7. Anxiety	0.38**	0.25**	0.36**	0.20**	0.37**	0.54**	–
Mean (SD)	11.94 (13.32)	3.09 (4.64)	4.07 (5.08)	1.33 (0.62)	4.15 (4.73)	12.82 (10.47)	10.52 (8.96)

**p < 0.01.

### Convergent Validity

TQ-R total score was significantly associated with Depression and Anxiety. All four subscales of TQ-R (Appearance, Social, Height, and Academic) had a significant positive correlation with Depression and Anxiety (Table 4).

## Discussion

The present study aimed to examine the psychometric properties of the Persian version of the TQ-R. Our results expand the previous research on the assessment of experiences of being teased. The results indicate that teasing is experienced in multiple domains.

Storch et al. (2004) defined five factors for the teasing structure, including Appearance, Social, Academic, Performance, and Family. However, contrary to our expectations, the five-factor model proposed by Storch et al. (2004) and Strawser et al. (2005) not supported in the current study. In addition, the three- and four-factor models (Faith et al., 2008) and the five-factor model (Gros et al., 2012) were not supported in this study. A review of the literature showed that the original five-factor model proposed by Storch et al. (2004) was not supported in any of the studies (Faith et al., 2008; Gros et al., 2012), and overall, there was a lack of consistency in the factor structure of the TQ.

One of the differences between the five-factor model proposed by Storch et al. (2004) and Strawser et al. (2005) and our analysis was eliminating the Performance and Family factors in our study. Being teased in the domains of Performance and Family may not be significant for adults. It is also interesting to point out that, in previous studies, these factors were non-significant (Storch et al., 2004; Faith et al., 2008; Gros et al., 2012).

However, the factors’ items in our final results were different to some extent compared with the primary model proposed by Storch et al. (2004). Our four-factor model had fewer items than the primary model, but the Appearance, Social, and Academic factors were the same as the primary model. In addition, in our results, a new factor, namely, Height, was proposed. This factor was proposed in the study of Gros et al. (2012). Finally, in our analysis, the Family factor was included in the Social factor.

Comparing the results of this study with Faith et al. (2008) and Gros et al. (2012) indicated similarities between the existing items in each of the factors, but in general, the arrangement of the factors was not the same. In the present study, six items are related to the Appearance factor. Examples of the items of this factor are as follows: “*I was teased because of some aspects of my appearance, like the way I dressed, wore glasses, and the color of my hair*,” and “*I was being teased because of the way I dress.*” In the five-factor model of Storch et al. (2004), elements of item 5 [“*I was teased because of ethnic and cultural differences* (*color of the skin, eating different food than other kids, wearing unique clothes, and so on*)”], item 6 (“*I was teased because the financial status of my family was not like the families of other kids*”), and item 29 (“*I was teased because of my funny name*”) were placed in the Family factor. However, in this study, they were included in the Appearance factor. This difference might be due to the fact that, in Iranian culture, most of the time, individuals experience teasing based on their physical and appearance features (Jamalvandia and Jamalvandib, 2016). Furthermore, the contents of the items included in the Family factor (items 5, 6, and 29) in the original version of the TQ-R (Storch et al., 2004) are mostly related to the Appearance factor in Iranian culture. For example, individuals experience teasing because of having a low financial status of the family (item 6) based on their appearance features (e.g., clothing). Moreover, in Iran, teasing victims mostly report teasing experiences in the domain of appearance.

The present study had eight items in the Social factor, such as “*I was teased because I was dissociable*” and “*since I was shy, I was teased.*” Item 22 (“*because I cried like a baby, I was teased*”) was in the Height factor, and item 25 (“*I was teased because I was paying more attention to the class than sports and other activities*”) was in the Social factor. However, they were deemed not relative to these factors, so they were transferred to Social and Academic factors, respectively.

The third factor in this study was “Height,” which included two items and was in line with the study of Gros et al. (2012). Below are examples of the items of the Height factor: “*I was teased because I was taller than other kids*;” “*I was teased because of my height.*” It is necessary to point out that item 26, “*I was teased because I wore eyeglasses*,” was moved from the Height factor to the Appearance factor.

The Academic factor consisted of five items. Some of these items are as follows: “*I was teased because I was my teacher’s pet*;” “*I was teased because I studied a lot.*” One of the more controversial items in this factor was item 21 (“*I was teased because I was more mature than the rest of the kids my age*”). Storch et al. (2004) and Faith et al. (2008) placed this item in the Appearance factor, but it was eliminated in the study of Gros et al. (2012) and the three-factor model of Faith et al. (2008). However, in this study, it was placed in the Academic factor and correlated with a relatively high factor load.

In the present study, the reported internal consistencies were higher than the previous studies (Storch et al., 2004; Faith et al., 2008; Gros et al., 2012) and displayed the excellent internal reliability of the Persian version of the TQ-R.

Finally, the significant positive correlation between TQ-R and its components with anxiety, depression, and distress supported the convergent validity of the TQ-R. Previous studies showed that teasing has a significant association with depression, anxiety, and distress (Szwimer et al., 2020).

Teasing is a common phenomenon in Iran’s schools and society. However, teasing experiences in Iran might be different from other nations because of cultural and educational differences. For example, while in most countries, students attend coeducational schools, Iranian students attend same-sex schools in which the frequency and the type of teasing experiences might be different from coeducational schools. Notwithstanding, because of the lack of a suitable measure for assessing teasing experiences in Iran, there has been no study on teasing in Iran, and our knowledge in this concern is too limited. We found only one related study that has been written from a sociological perspective and in which authors studied the application of nicknames among Ilami Kurdish students in West of Iran (Jamalvandia and Jamalvandib, 2016). Accordingly, the nickname concept is common among Ilami speakers, which inspired researchers to study this sociolinguistic phenomenon. The authors concluded that high school students in Iran configure most of the nicknames based on their peers, friends, and classmates’ physical and appearance features (Jamalvandia and Jamalvandib, 2016). All in all, future studies are needed to use a suitable teasing measure for studying teasing experiences in Iran’s society and possible cross-cultural differences.

This study had a few limitations. First, because this research was a cross-sectional study, it could not measure a teasing experience entirely and correctly because the passage of time can deteriorate or augment the recollection of a teasing experience. As with all self-report measures, data based on the recall can raise questions of reliability. Thus, longitudinal research is recommended for future studies. The second limitation was that we did not use a similar measure to evaluate TQ-R’s criterion validity. This shortcoming was because of the lack of accessible and reliable tools in the Persian language to measure teasing experiences. We recommend using different measurement methods and not relying solely on self-report scales. Finally, since the factor structure of TQ-R obtained from the EFA, it is recommended to conduct a CFA on another similar sample in the future studies.

According to our findings, we can consider the TQ with 23 items and four factors of Appearance, Social, Height, and Academic as a proper tool for measuring teasing experiences. The importance of teasing experiences has been shown in a wide range of psychopathologies (Stitt et al., 2015; Lund and Ross, 2017), and TR-Q is an excellent scale for measuring multiple domains of teasing experiences. Overall, the Persian version of the TQ (TQP-23) can be widely used in psychological studies of disorders like depression, anxiety, and others in which teasing experiences have played a role.

## Data Availability Statement

The raw data supporting the conclusions of this article will be made available by the authors, without undue reservation.

## Ethics Statement

The studies involving human participants were reviewed and approved by the Ethical Committee of the Iran University of Medical Sciences. Written informed consent to participate in this study was provided by the participants’ legal guardian/next of kin.

## Author Contributions

AE and MEA: designed the study and drafted the manuscript. HK: performed data analysis. MHS and EFS: reviewed and revised the manuscript.

## Conflict of Interest

The authors declare that the research was conducted in the absence of any commercial or financial relationships that could be construed as a potential conflict of interest.

## References

[B1] Beck A. T. Epstein N. Brown G. Steer R. A. (1988a). An inventory for measuring clinical anxiety: psychometric properties. *J. Consult. Clin. Psychol.* 56 893–897. 10.1037//0022-006x.56.6.893 3204199

[B2] Beck A. T. Steer R. A. Carbin M. G. (1988b). Psychometric properties of the beck depression inventory: twenty-five years of evaluation. *Clin. Psychol. Rev.* 8 77–100. 10.1016/0272-7358(88)90050-5

[B3] Benas J. S. Gibb B. E. (2011). Childhood teasing and adult implicit cognitive biases. *Cogn. Ther. Res.* 35 491–496. 10.1007/s10608-010-9326-y

[B4] Du Toit M. Du Toit S. H. C. Hawkins D. M. (2001). *Interactive LISREL: User’s Guide.* Lincolnwood, IL: Scientific Software International.

[B5] Epstein J. Santo R. M. Guillemin F. (2015). A review of guidelines for cross-cultural adaptation of questionnaires could not bring out a consensus. *J. Clin. Epidemiol.* 68 435–441. 10.1016/j.jclinepi.2014.11.021 25698408

[B6] Faith M. A. Storch E. A. Roberti J. W. Ledley D. R. (2008). Recalled childhood teasing among non-clinical, non-college adults. *J. Psychopathol. Behav. Assess.* 30 171–179. 10.1007/s10862-007-9062-0

[B7] Gadekar U. (2016). Eve teasing and its psychosocial influence among the adolescent girls. *Int. J. Curr. Adv. Res.* 5 1028–1031.

[B8] Gregg D. H. Somers C. L. Pernice-Duca F. Van Dale K. G. (2016). Teasing experiences and risk-taking: gender and self-esteem as moderator and mediator. *J. Sch. Viol.* 15 365–385. 10.1080/15388220.2015.1054935

[B9] Gros D. F. Gros K. S. McCabe R. E. Antony M. M. (2012). Psychometric evaluation of the factor structure of the Teasing Questionnaire–Revised (TQ-R). *J. Psychopathol. Behav. Assess.* 34 542–551. 10.1007/s10862-012-9301-x

[B10] Hu L. Bentler P. M. (1999). Cutoff criteria for fit indexes in covariance structure analysis: conventional criteria versus new alternatives. *Struct. Equ. Model.* 6 1–55. 10.1080/10705519909540118

[B11] Jamalvandia B. Jamalvandib A. (2016). Application of Nicknames Among Ilami Kurdish Students, West of Iran. *Sociology* 6 604–614.

[B12] Kaviani H. Mousavi A. (2008). Psychometric properties of the Persian version of Beck Anxiety Inventory (BAI). *Tehran Univ. Med. J.* 66 136–140.

[B13] Keltner D. Capps L. Kring A. M. Young R. C. Heerey E. A. (2001). Just teasing: a conceptual analysis and empirical review. *Psychol. Bull.* 127 229–248. 10.1037/0033-2909.127.2.229 11316012

[B14] Kline R. B. (2015). *Principles and Practice of Structural Equation Modeling.* New York, NY: Guilford publications.

[B15] Kostanski M. Gullone E. (2007). The impact of teasing on children’s body image. *J. Child Fam. Stud.* 16 307–319. 10.1007/s10826-006-9087-0

[B16] Kruger J. Gordon C. L. Kuban J. (2006). Intentions in teasing: When” just kidding” just isn’t good enough. *J. Per. Soc. Psychol.* 90 412–425. 10.1037/0022-3514.90.3.412 16594828

[B17] Lund E. M. Ross S. W. (2017). Bullying perpetration, victimization, and demographic differences in college students a review of the literature. *Trauma Violence Abuse* 18 348–360. 10.1177/1524838015620818 26759417

[B18] Manian N. Schmidt E. Bornstein M. H. Martinez P. (2013). Factor structure and clinical utility of BDI-II factor scores in postpartum women. *J. Affect. Disord.* 149 259–268. 10.1016/j.jad.2013.01.039 23521870 PMC3672272

[B19] McCabe R. E. Antony M. M. Summerfeldt L. J. Liss A. Swinson R. P. (2003). Preliminary examination of the relationship between anxiety disorders in adults and self-reported history of teasing or bullying experiences. *Cogn. Behv. Ther.* 32 187–193. 10.1080/16506070310005051 16291550

[B20] McCabe R. E. Miller J. L. Laugesen N. Antony M. M. Young L. (2010). The relationship between anxiety disorders in adults and recalled childhood teasing. *J. Anxiety Disord.* 24 238–243. 10.1016/j.janxdis.2009.11.002 19963339

[B21] Meyers L. S. Gamst G. C. Guarino A. (2013). *Performing Data Analysis Using IBM SPSS.* Hoboken, NJ: John Wiley & Sons.

[B22] Muris P. Littel M. (2005). Domains of childhood teasing and psychopathological symptoms in Dutch adolescents. *Psychol. Rep.* 96 707–708. 10.2466/pr0.96.3.707-708 16050627

[B23] Mutlu B. Yilmaz M. (2018). Child-adolescent teasing scale: psychometric properties of the Turkish version. *Child Adolesc. Ment. Health* 23 283–290. 10.1111/camh.12250 32677296

[B24] Roth D. A. Coles M. E. Heimberg R. G. (2002). The relationship between memories for childhood teasing and anxiety and depression in adulthood. *J. Anxiety Disord.* 16 149–164. 10.1016/s0887-6185(01)00096-2 12194541

[B25] Schumacker R. E. Lomax R. G. (2015). *A Beginner’s Guide to Structural Equation Modeling.* New York, NY: Routledge.

[B26] Stefan-Dabson K. Mohammadkhani P. Massah-Choulabi O. (2007). Psychometrics characteristic of Beck Depression Inventory-II in patients with magor depressive disorder. *Arch. Rehabil.* 29 82–88.

[B27] Stitt N. Francis A. J. Field A. M. Carr S. N. (2015). Positive association between reported childhood peer teasing and adult borderline personality disorder symptoms. *J. Child Adolesc. Trauma* 8 137–145. 10.1007/s40653-015-0045-0

[B28] Storch E. A. Ledley D. R. (2005). Peer victimization and psychosocial adjustment in children: current knowledge and future directions. *Clin. Pediatr.* 44 29–38. 10.1177/000992280504400103 15678228

[B29] Storch E. A. Roth D. A. Coles M. E. Heimberg R. G. Bravata E. A. Moser J. (2004). The measurement and impact of childhood teasing in a sample of young adults. *J. Anxiety Disord.* 18 681–694. 10.1016/j.janxdis.2003.09.003 15275946

[B30] Strawser M. S. Storch E. A. Roberti J. W. (2005). The Teasing Questionnaire—Revised: measurement of childhood teasing in adults. *J. Anxiety Disord.* 19 780–792. 10.1016/j.janxdis.2004.09.005 16076424

[B31] Szwimer E. Mougharbel F. Goldfield G. S. Alberga A. S. (2020). The association between weight-based teasing from peers and family in childhood and depressive symptoms in childhood and adulthood: a systematic review. *Curr. Obes. Rep.* 9 15–29. 10.1007/s13679-020-00367-0 32002762

[B32] Thompson J. K. Cattarin J. Fowler B. Fisher E. (1995). The perception of teasing scale (POTS): a revision and extension of the physical appearance related teasing scale (PARTS). *J. Pers. Assess.* 65 146–157. 10.1207/s15327752jpa6501_11 16367650

[B33] Thompson J. K. Fabian L. J. Moulton D. O. Dunn M. E. Altabe M. N. (1991). Development and validation of the physical appearance related teasing scale. *J. Pers. Assess.* 56 513–521. 10.1207/s15327752jpa5603_12 1865309

[B34] Tian-mei Z. (2009). Preliminary reliability & validity assessment of the teasing questionnaire in Chinese version among Chinese Middle School Students. *J. Neijiang Norm. Univ.* 24 63–67.

[B35] Watanabe P. I. Fontana F. E. Choi S.-I. da Silva M. P. Mazzardo O. Waldron J. (2019). Development and validation of the Brazilian Weight Teasing during Physical Activity Scale. *Rev. Bras. Ciênc. Mov.* 27 70–80. 10.31501/rbcm.v27i1.7697

[B36] Zhou T. Luo L. (2015). The relationship between parental rearing patterns and teenagers teasing. *Psychology* 6 1456–1468. 10.4236/psych.2015.612143

[B37] Zlomke K. Jeter K. Cook N. (2016). Recalled childhood teasing in relation to adult rejection and evaluation sensitivity. *Pers. Individ. Dif.* 89 129–133. 10.1016/j.paid.2015.10.021

[B38] Zuba A. Warschburger P. (2017). The role of weight teasing and weight bias internalization in psychological functioning: a prospective study among school-aged children. *Eur. Child. Adolesc. Psychiatry* 26 1245–1255. 10.1007/s00787-017-0982-2 28361259

